# High-Density Energetic Materials with Low Mechanical Sensitivity and Twinning Derived from Nitroimidazole Fused Ring

**DOI:** 10.3390/molecules29020353

**Published:** 2024-01-10

**Authors:** Yaxin Liu, Meifang Lv, Guofeng Zhang, Zhen Dong, Zhiwen Ye

**Affiliations:** School of Chemistry and Chemical Engineering, Nanjing University of Science and Technology, Nanjing 210094, China; 15150936191@163.com (Y.L.); lvmf@njust.edu.cn (M.L.); 321103010132@njust.edu.cn (G.Z.); dongzhen90@126.com (Z.D.)

**Keywords:** high density, low mechanical sensitivity, twinning, fused ring, nitroimidazole

## Abstract

The innovative synthesis of 3,8-dibromo-2,9-dinitro-5,6-dihydrodiimidazo [1,2-a:2′,1′-c]pyrazine and 3,9-dibromo-2,10-dinitro-6,7-dihydro-5*H*-diimidazo [1,2-a:2′,1′-c][1,4]diazepine is described in this study. The tricyclic fused molecular structures are formed by the respective amalgamation of piperazine and homopiperazine with the imidazole ring containing nitro. Compound **1** and **2** possess excellent high-density physical properties (ρ_1_ = 2.49 g/cm^3^, ρ_2_ = 2.35 g/cm^3^) due to the presence of a fused ring structure and Br atom. In addition to their high density, they have high decomposition temperatures (T_d_ > 290 °C) which means that they have excellent thermal stability and can be used as potential heat-resistant explosives. Low mechanical sensitivities (IS > 40 J, FS > 360 N) are observed. The twinning structure of 2 was resolved by X-ray diffraction. Non-covalent interaction analysis, Hirshfeld surfaces, 2D fingerprint plot, and Electrostatic potential analysis were used to understand the intramolecular interactions in relation to physicochemical properties. The unique structures of this type of compound provide new potential for the evolution of energetic materials.

## 1. Introduction

Because energetic materials possess explosive groups like nitro groups or oxidizers and combustibles, chemical reactions can be carried out independently. After three hundred years of dynamic development, there has been sustained research activity showing that energetic materials are moving towards high reliability, safety, and density. Imidazole energetic compounds have large π bonds with electron-delocalized domains and conjugated aromaticity that are the sources of their stability. They have a high enthalpy of formation and have become a typical class of compounds in energetic materials [1,2]. Imidazole rings have limited modification sites and little structural variability. Nitro-containing imidazoles are common frameworks for energetic materials. Due to the electron-withdrawing effect of the nitro group, N-H in nitroimidazoles exhibits acidity, and hydrogen atoms tend to drop off to form nucleophilic reagents.

Low mechanical sensitivity and high thermal stability are essential safety indicators for energetic materials. The physical properties of a high density can secure the application of energetic materials. There has been an increased recognition that more attention needs to be paid to the combination of low mechanical sensitivity, high thermal stability, and high density. Energetic materials with fused rings, a unique class of large conjugated structures containing two or more rings sharing two atoms and bonds between the rings, have been identified as promising contenders to traditional energetic materials [3]. Fused ring energetic compounds are important skeletons for the future design of novel energetic compounds due to their conjugated planar structures that can effectively increase the density and molecular stability of the compounds with good thermal stability and low sensitivity [4]. The appearance of fused rings and the increase in the number of rings affect the value of the electrostatic potential in the central region of the surface of the explosive molecule, which is related to the sensitivity of the energetic materials [5]. They improve the safety of the synthesis, transfer, and storage of energetic materials. The introduction of heteroatoms is a way to increase the density of energetic materials. The Br element occurs rarely in the field of energetic materials and has a particular scarcity. Each introduction of the Br element has a positive impact on increasing the density of the compounds, e.g., 5-amino-1*H*-tetrazolium bromide monohydrate (5-AtHBr) has a density of more than 2.00 g/cm^3^, which is higher compared to the parent 5-AT [6].

Twinning is defined as two crystals or two parts of a crystal that form a mirror-symmetric orientation relationship along a common crystal plane (i.e., a specific orientation relationship) [7,8,9]. Twinning is still an area of active research, and metallic elements are always involved in twinning [10,11,12]. The two crystals or two parts of a crystal are called twinnings, and the joint crystal surface is called the twinning–crystal plane. The formation of twinnings is closely related to the layer dislocation energy. Crystals with high-layer dislocation energy are less likely to produce twinnings. Depending on the cause of the formation, twinnings can be categorized as deformation, growth, or annealing twinnings.

In this paper, fused ring compound 3,8-dibromo-2,9-dinitro-5,6-dihydrodiimidazo [1,2-a:2′,1′-c]pyrazine and 3,9-dibromo-2,10-dinitro-6,7-dihydro-5*H*-diimidazo [1,2-a:2′,1′-c][1,4]diazepine containing dinitro groups were synthesized. This study sets out to explore the compounds with unique fused ring structures, high density, high safety, and thermal stability. The crystal grown from 3,9-dibromo-2,10-dinitro-6,7-dihydro-5*H*-diimidazo [1,2-a:2′,1′-c][1,4]diazepine was a growth twinning. The internal structure was determined by X-ray diffraction. The molecular interaction forces were explored through non-covalent interaction analysis, and electrostatic potential analysis was employed to observe the potential distribution of the compound.

## 2. Results and Discussion

### 2.1. Synthesis

**Scheme 1:**
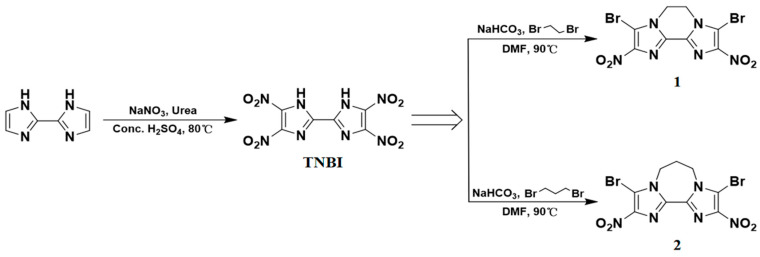
Synthesis of compound **1** and **2**.

Sodium nitrate as the nitrifying agent and urea as the catalyst were added to the solution formed by pure biimidazole in excessive concentrated sulfuric acid. Nitrification occurs at high temperatures. 4,4′,5,5′-Tetranitro-1*H*,1′*H*-2,2′-biimidazole (TNBI) was precipitated in ice water when the reaction was completed. The strong base sodium bicarbonate reaction with TNBI at room temperature resulted in the formation of energy-containing anions by the falling off of the active hydrogen on the imidazole in TNBI. Subsequently, either 1,2-dibromoethane or 1,3-dibromopropane was added as the attacking reagent in the substitution reaction. They are carried out with TNBI for the ring closure reaction and the substitution of bromine atoms for nitro in parallel. The tricyclic cross-linking and substitution reactions were carried out simultaneously at high temperatures for 12 h (Scheme 1). Based on the hydrophobicity of the products, the precipitated products were obtained by pouring the reaction mixture solution into a large amount of ice water. The final product of 3,8-dibromo-2,9-dinitro-5,6-dihydrodiimidazo[1,2-a:2′,1′-c]pyrazine (**1**) and 3,9-dibromo-2,10-dinitro-6,7-dihydro-5*H*-diimidazo[1,2-a:2′,1′-c][1,4]diazepine (**2**) were obtained by the purification of the precipitates in 72.6% and 80.2% yields, respectively.

### 2.2. Spectral Analyses

The ^1^H and ^13^C NMR spectra of compound **1** and **2** were documented on a Bruker 500 MHz digital NMR spectrometer functioning at 500 MHz and 126 MHz, respectively. All NMR spectra are presented in the supporting information. The NMR spectra were plotted using the deuterated DMSO as the solvent for the locked field. The ^1^H signal for compound **1** is the proton signal on the saturated alkyl group in piperazine on the fused ring, which is at 4.59 ppm. The ^1^H signal for compound **2** is the proton signal on the saturated alkyl group in homopiperazine on the fused ring. Due to the symmetry of compound **2**, its proton signal on the middle sec-carbon atom produces a quintuple peak at 2.57 ppm, and their coupling constant is 5.9 Hz. The proton signals of the other sides of the saturated sec-carbons are between 4.41 ppm and 4.35 ppm. The ^13^C signal of the non-common side on the piperazine of compound **1** is 42.8 ppm, while the ^13^C signal of the carbon atom that the piperazine shares with the imidazole is 144.9 ppm. The ^13^C signal of the carbon attached to the nitro functional group on the imidazole ring is at 134.4 ppm, and the ^13^C signal of the carbon attached to the Br atom is at 109.1 ppm. The ^13^C signal of the middle sec-carbon of homopiperazine on compound **2** is at 25.07 ppm, and the ^13^C signals of the other non-common side carbon atoms are at 47.99 ppm. The ^13^C signal of the C atom that homopiperazine shares with imidazole is at 144.41 ppm. The ^13^C signal of the C atom attached to the nitro functional group on the imidazole is 136.0 ppm, and the ^13^C signal of the C atom attached to the bromine atom is 109.7 ppm.

In addition, Thermo Fourier infrared spectroscopy mapped the infrared absorption spectra of compound **1** and **2** (visible in the supporting information). The characteristic peak of absorption of saturated alkanes on piperazine in the infrared spectrum of compound **1** is 2988.73 cm^−1^, and the characteristic peaks of absorption of saturated alkanes on homopiperazine in the infrared spectrum of compound **2** are 2988.22 cm^−1^ and 2900.60 cm^−1^. The IR spectra of compound **1** and **2** showed characteristic peaks of antisymmetric and symmetric telescopic absorption of nitro groups in the range of 1650 to 1500 cm^−1^ and 1370 to 1250 cm^−1^, respectively. The bromine atoms contained in the compounds are reflected in the characteristic absorption peaks in the 600 cm^−1^ to 475 cm^−1^ region of the infrared spectra.

### 2.3. Crystallographic Analyses

An X-ray crystal was obtained by the slow evaporation of saturated ethyl acetate and DMF solution of compound **2** to understand the detailed structure inside the compound. The measurable translucent needle crystals with the size of 0.220 mm × 0.020 mm × 0.020 mm of compound **2** were evaluated by XRD (X-ray diffraction). The refinement information of the crystal data of compound **2** is shown in Table 1, and other crystal information consisting of bond lengths, bond angles, and hydrogen bonds is visible in the Supplementary Material.

**Figure 1:**
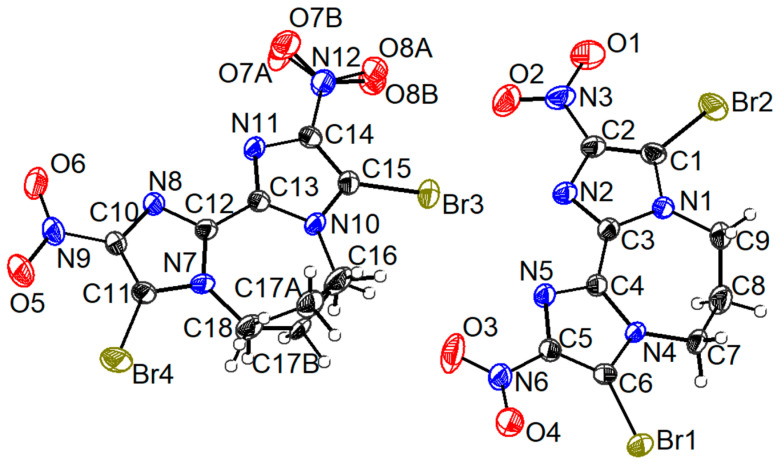
Molecular structure of compound **2** (50% thermal ellipsoid plot).

Compound **2** crystallizes in the monoclinic lattice space group P2_1_/c, and the structure of the twinning compound is shown in Figure 1. There are eight molecules in each unsymmetrical unit cell (Z = 8). The calculated density of compound **2** is 2.216 g/cm^3^ at 296 K. The bond lengths (N1-C3: 1.375 (4) Å, C3=N2: 1.315 (4) Å, N2-C2: 1.357 (4) Å, C2=C1: 1.369 (4) Å, C1-N1: 1.373 (4) Å, N4-C4: 1.382 (4) Å, C4=N5: 1.313 (4) Å, N5-C5: 1.361 (4) Å, C5=C6: 1.365 (4) Å, C6-N4: 1.374 (4) Å) on the imidazoles in the molecules of both configurations are more evenly balanced compared to the standard bond lengths, indicating their good conjugated aromaticity. As two of the fused rings, the imidazoles all have bond angles (N1-C1-C2: 104.3 (2)°, C2-N2-C3: 103.7 (2)°, N4-C6-C5: 105.0 (2)°, C5-N5-C4: 104.1 (2)°, N7-C11-C10: 105.1 (2)°, C10-N8-C12: 104.5 (2)°) very close to those of the standard five-membered ring, and their torsion angles (N1-C1-C2-N2: −0.2 (3)°, N4-C6-C5-N5: −0.3 (319)°, N10-C15-C14-N11: −0.3 (321)°, N7-C11-C10-N8: 0.1 (321)°) express their planarity. The seven elemental heterocyclic in the bridged ring demonstrates the specificity of the twinning structure, and the seven elemental heterocyclic in the molecular configuration that does not generate different molecular orientations has bond angles (C7-C8-C9: 114.6 (3)°) and bond lengths (C7-C8: 1.495 Å, C8-C9: 1.502 Å). The seven elemental ring with different molecular orientations has bond angles (C16-C17A-C18: 120.9 (5)°, C16-C17B-C18: 122.9 (8)°) and bond lengths (C16-C17A:1.430 Å, C17A-C18: 1.471 Å, C16-C17B:1.392 Å).

**Figure 2:**
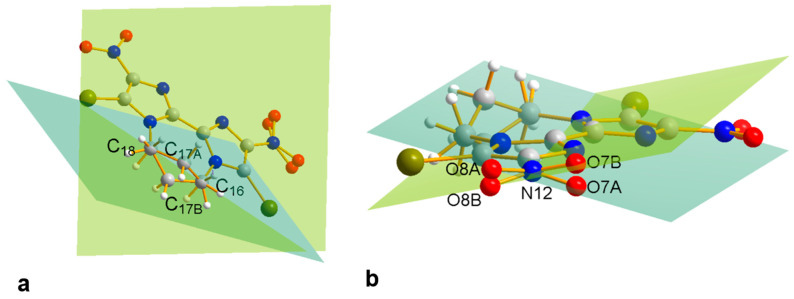
(**a**) Dihedral angles formed by different growth orientations of alkyl group in compound **2**. (**b**) Dihedral angles formed by different growth orientations of nitro functional group in compound **2**.

**Figure 3:**
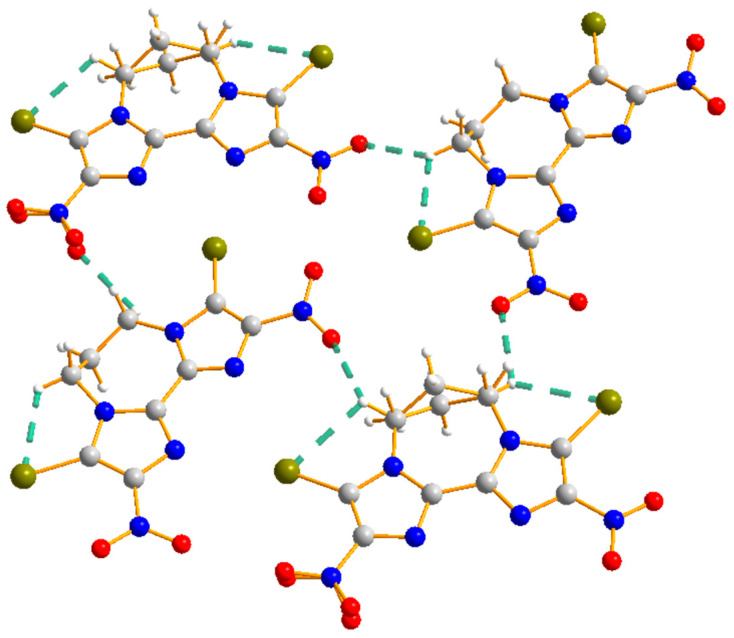
Hydrogen bonds in compound **2** molecules.

**Figure 4:**
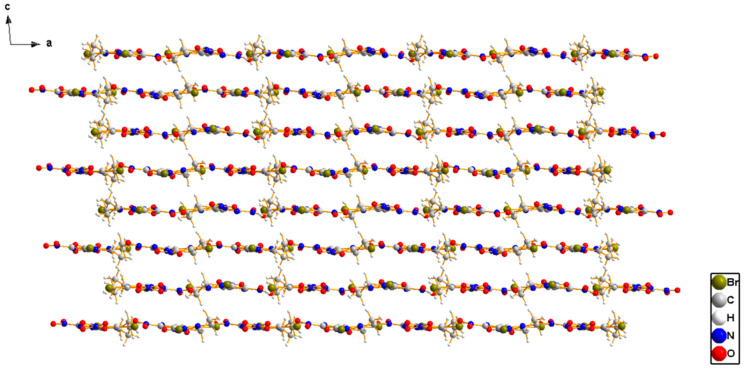
Crystal packing system and hydrogen bond interactions of compound **2** (b-axis).

Meanwhile, the dihedral angle of the plane of O7A-N12-O8A and the plane of O7B-N12-O8B, produced by different growth orientations of the oxygen atoms on the nitro group, is 26.8°, and the dihedral angles of plane: C15-C17A-C16 and plane: C15-C17B-C16, produced by different growth orientations of the C17 atom on the alkyl bridge, are 68.0°. Their occurrence indicates the structural features of twinning crystals (Figure 2). Figure 3 demonstrates the presence of extensive hydrogen bonding within the crystal. Neighboring planar layers are ordered in opposite directions and stacked together in the crystal space (b axial view) in Figure 4.

### 2.4. Analyses of the Electronic Properties

#### 2.4.1. Non-Covalent Interaction Analysis

**Figure 5:**
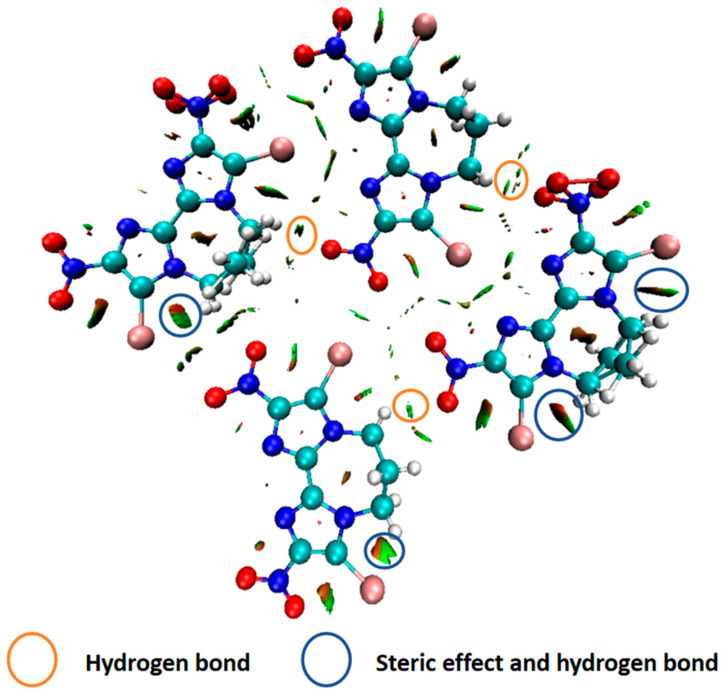
Non-covalent interaction analysis for compound **2**.

In order to investigate the molecular interactions, non-covalent interaction (NCI) analysis was performed on compound **2** with the Multiwfn v3.8 and VMD v1.9.2 [13,14,15,16,17]. The NCI analysis of the compound is illustrated in Figure 5. Red electron stacks representing site-blocking effects are present within the seven-membered ring and the imidazoles. There are some blue-green dotted high-density electron stacks representing hydrogen bonding generation between the hydrogen and oxygen atoms. The one-half orange and one-half green color of the isosurfaces near the bromine atoms represents the combination of site-blocking effects and hydrogen bonding. Halogen bonds can be observed between Br and N atoms (N from the imidazole ring) and between Br and O atoms. Halogen bonds are weak intermolecular interactions similar to hydrogen bonding and have a wide range of applications in many fields, such as molecular recognition and crystal engineering. They can have a positive effect on the design of new energetic materials with reduced susceptibility to explosions [18]. The extensive hydrogen bonding and interaction forces in the compound suggest stability.

#### 2.4.2. Hirshfeld Surface Analysis

**Figure 6:**
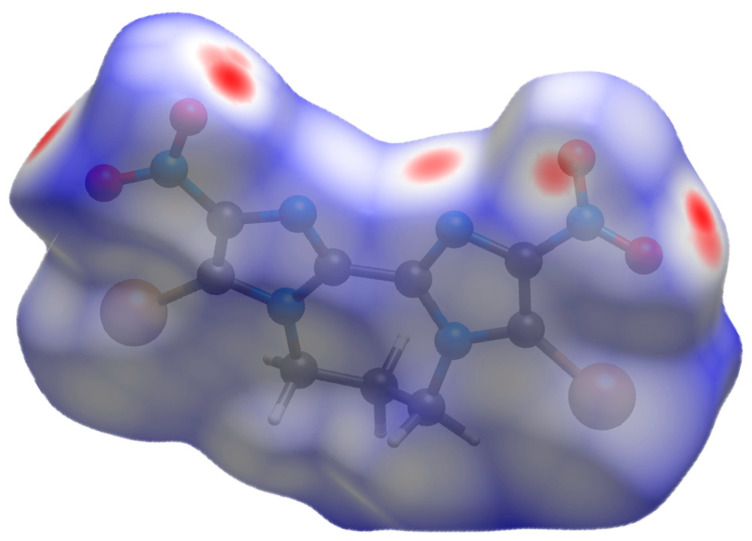
Hirshfeld surfaces of compound **2**.

To successfully separate the electron density of the molecule into molecular fragments, the Hirshfeld surface defines the space occupied by the molecule. The different colors on the Hirshfeld surface represent different electron densities and regions of atomic contact. Red areas represent high electron density and hydrogen bonds. Blue areas represent low electron densities and little interaction. As illustrated in Figure 6, the Hirshfeld surface of compound **2** has red areas around the edges, indicating the formation of hydrogen bonds in the molecule.

**Figure 7:**
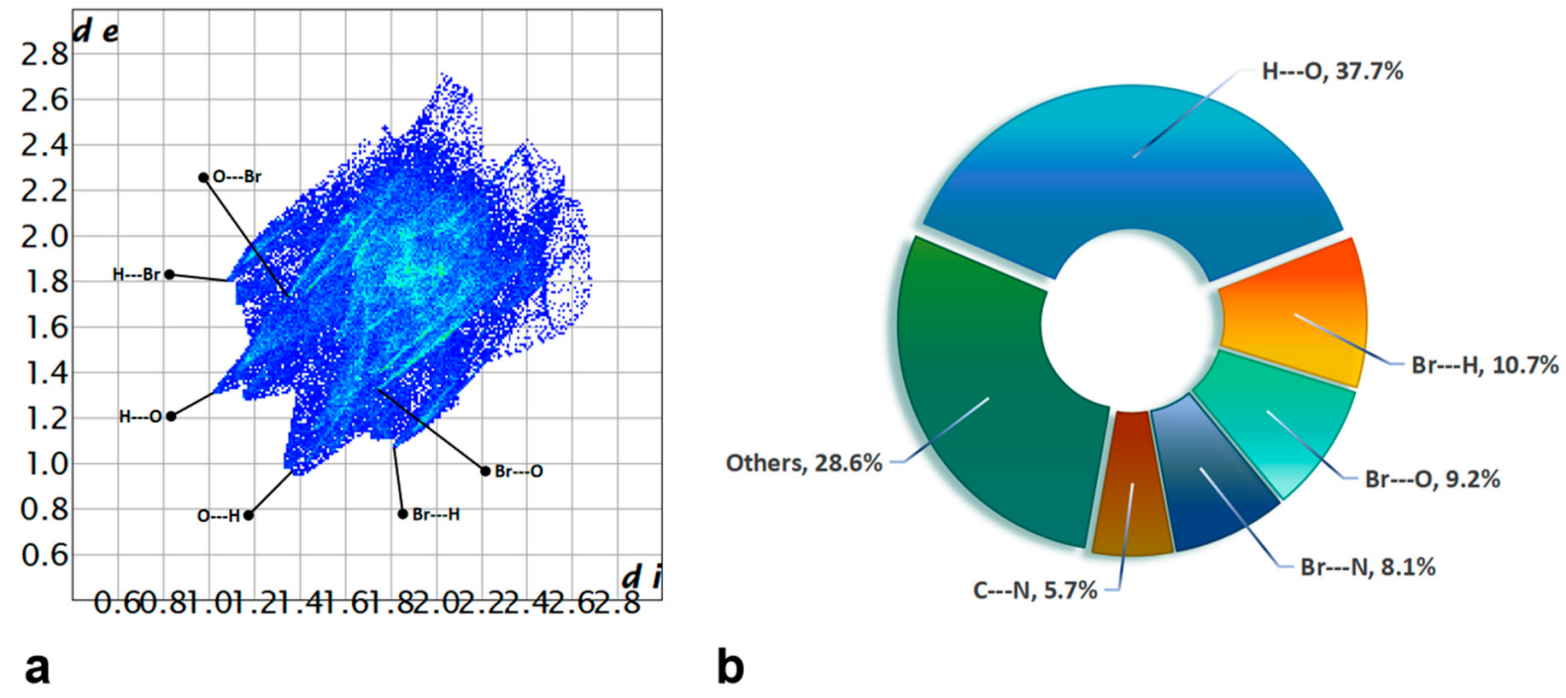
(**a**) 2D Fingerprint plot of compound **2**. (**b**) Individual atomic contact percentage contribution of compound **2**.

The 2D fingerprint plot drawn using the software CrystalExplorer 17 [19,20] is presented in Figure 7a, and the atomic contact ratio percentage plot derived based on the data is shown in Figure 7b. As can be seen from the plots, the H---O contact pair and Br---H contact pair possess 37.7% and 10.7% contact percentages, respectively, which confirm that these two hydrogen bond contact pairs have the highest atomic contact ratio. Those data confirm that H---O and Br---H hydrogen bond contacts are essential forces in the interaction and support of low mechanical sensitivities. In addition to these hydrogen bonds, other interactions are reflected in the different contact pairs in these plots. Halogen bonds are also reflected in the 2D fingerprint plot and atomic contact percentage contribution plot. The proportion of Br---O contact pairs (9.2%) is higher than that of Br---N (8.3%).

#### 2.4.3. Electrostatic Potential Analysis

**Figure 8:**
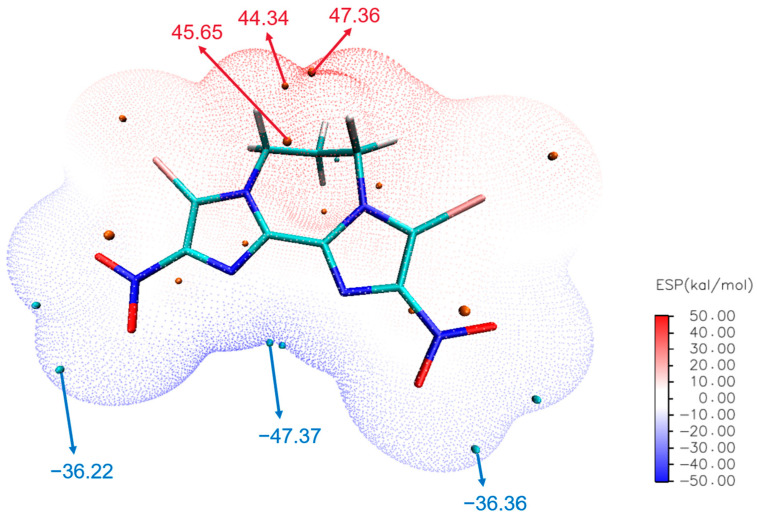
Electrostatic potential diagram of compound **2**.

The electrostatic potential diagram [21] of compound **2** is plotted in Figure 8 to understand the potential distribution of the compounds. The blue region represents the negative potential region, and the red region represents the positive potential region. The white region represents the potential that tends to zero. The positive potential regions of compound **2** are concentrated near the seven-membered ring. The positive values of the electrostatic potential above the front region of the bromine atoms and maximum value around the bromine atoms are 34.02 and 34.20 kal/mol, respectively. The negative potential regions are concentrated near the nitro group. Values of electrostatic potentials above the central regions of the molecular surface are strongly related to the sensitivities of energetic molecules [18]. The electrostatic potential above the center region of the molecular surface is in the white region in the figure, which indirectly supports its low mechanical sensitivity. In energetic compounds, more extensive areas with positive potentials usually result in increased impact sensitivities [22]. The highest positive potential (47.36 kal/mol) almost coincides with the absolute value of the lowest negative potential (−47.37 kal/mol), and the positive and negative distribution of the potential area region is more uniform (positive potential area percentage: 39.2%; negative potential area percentage: 60.8%). A lower percentage of the positive potential region and the uniform potential distribution benefit the formation of low mechanical sensitivity [23]. 

### 2.5. Physicochemical and Explosive Properties

**Figure 9:**
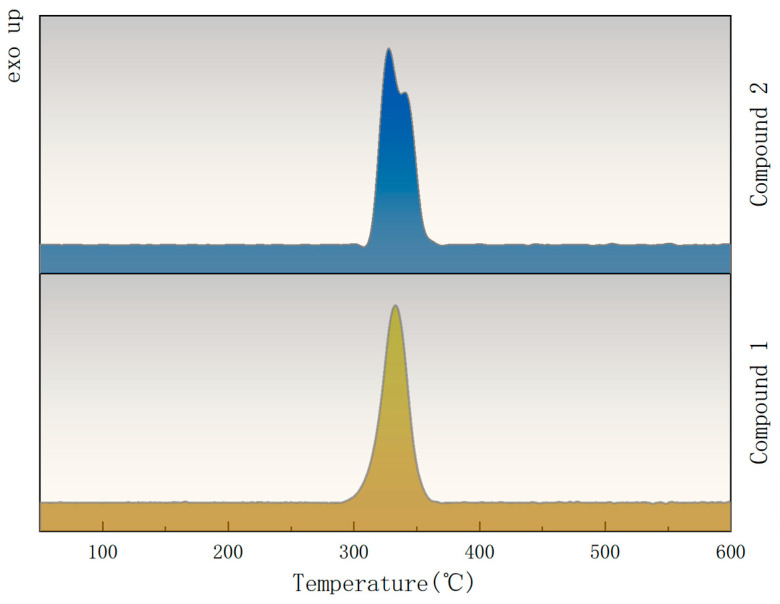
Differential scanning calorimetry of compound **1** and **2**.

The thermostability of compound **1** and **2** was measured by the differential scanning calorimetry (DSC) to explore their applications. It is an analytical method that measures the energy difference between the specimen and the reference as a function of temperature under programmed temperature increase. The tested compounds were heated in an aluminum trioxide crucible at 5.0 K/min under nitrogen protection with a nitrogen flow rate of 50 mL/min. The onset of decomposition temperatures of compound **1** and **2** are 292.4 °C and 311.3 °C (Figure 9), respectively. They are higher than the onset of decomposition temperature of 290 °C for trinitrotoluene (TNT) [24,25], a conventional explosive. The peak decomposition temperatures of compound **1** and **2** were 332.8 °C and 328.2 °C, respectively. The high thermal stability gives them the potential to become thermally resistant explosives.

In addition to excellent thermal stability, the compounds have excellent mechanical sensitivity. The impact and friction sensitivities of the compounds are obtained by using the BAM Fallhammer and BAM Friction Tester at room temperature. Compound **1** and **2** have an impact sensitivity of more than 40 J and a friction sensitivity of more than 360 N (Table 2), which is better than TNT (IS = 15 J, FS = 353 N). Their low mechanical sensitivity ensures their high safety and ease of transportation.

Because of the dense existence of Br atoms and fused rings, they exhibit unusual levels of over 2.00 g/cm^3^ (ρ_1_ = 2.49 g/cm^3^, ρ_2_ = 2.35 g/cm^3^) in density measured by a gas pycnometer at 25 °C. The Gaussian 09W [26] made calculations of the heat of generation via isodesmic reactions (see Supporting Information for details). The positive heat of generation for compound **1** and **2** were calculated to be 0.82 and 0.75 kJ/g, respectively. The resulting detonation velocities in the normal range (1: 6.637 km/s, 2: 6.472 km/s) were calculated using EXPLO5 6.01 [27] and are higher than those of conventional explosive DADP [24] (6.246 km/s). The detonation pressure of compound **1** (20.6 GPa) is higher than that of TNT (19.5 GPa), and the detonation pressure of compound **2** is 19.1 GPa.

## 3. Materials and Methods

All solvents and base chemical materials above AR purity were purchased regularly without further purification. All chemical materials required in this work are potential precursors to energetic compounds, and they can result in explosions when the experiment is in progress if impact, friction, or high pressure is encountered. It is strongly recommended that appropriate safety precautions, such as safety masks, goggles, and nitrile gloves, should consistently be implemented.

1. 4,4′,5,5′-tetranitro-1*H*,1′*H*-2,2′-biimidazole (TNBI) [28].

To an 80 mL aqueous solution of 18.30 g sodium bisulfate (175.50 mmol, 4.6 eq), 50 mL of ethanol and 12.77 g (38 mmol, 1.0 eq) of 40% glyoxal solution were added and stirred for 2 h at room temperature. The resulting white solid was then filtered and washed with ethanol and ether. The solid was redissolved in aqueous ammonia solution (138 mL, 25%) and ammonium carbonate (5.0 g). The mixture was refluxed for 4 h and then cooled to room temperature. The solid was filtered out and washed with ethanol, then oven-dried to give the light gray product bimidazole (1.18 g, 22.9%). Precisely 1.10 g (8.25 mmol, 1.0 eq) of bimidazole was slowly added to 12 mL of excess 98% concentrated sulfuric acid at 30 °C with vigorous stirring for 1 h. After waiting for the complete dissolution of the bimidazole in the sulfuric acid, 0.05 g (0.1 eq) of urea was added as a catalyst. The temperature of the mixture was controlled at about 30 °C, and 4.15 g of sodium nitrate (48.85 mmol, 1.5 eq) was added to the mixture in portions. The temperature was slowly increased to 80 °C and reacted for 5 h. The reaction was accompanied by the production of dark brown gas. At the end of the reaction, the reaction solution was cooled to room temperature and poured into 500 mL of ice water. The mixture was filtered, and the precipitated product was washed with a small amount of ice water. The bright yellow product was obtained by natural drying (1.63g, 62.9%).

2. 3,8-dibromo-2,9-dinitro-5,6-dihydrodiimidazo [1,2-a:2′,1′-c]pyrazine (**1**).

Sodium bicarbonate (0.17 g, 2.0 mmol) was added to a solution of TNBI (0.314 g, 1.0 mmol) in DMF (5 mL). The reaction mixture was stirred at room temperature for 15 min. Then, 1,2-dibromoethane (0.19 g, 1.0 mmol) was added and heated at 90 °C with continuous stirring for 12 h. After cooling to ambient temperature, the reaction mixture was poured into ice water (100 mL), and the precipitate was collected and washed with ice water (20 mL). Finally, the product was obtained by recrystallization with acetonitrile. (0.29 g, 71.6%) ^1^H NMR (500 MHz, DMSO): δ = 4.59 (s, 4H). ^13^C NMR (126 MHz, DMSO): 144.9, 134.4, 109.1, 42.8. IR (KBr pellet): 2988.73, 1743.94, 1615.34, 1521.53, 1472.43, 1449.33, 1407.18, 1332.77, 1316.82, 1280.41, 1240.34, 1222.61, 1170.69, 1044.94, 986.01, 925.25, 847.72, 825.08, 758.12, 727.5, 664.06, 630.38, 574.35, 489.82 cm^−1^. Elemental analysis for C_8_H_4_Br_2_N_6_O_4_ (405.87). Calculated value (%): C 23.55, H 0.99, N 20.60. Measured value (%): C 23.58, H 0.96, N 20.62.

3. 3,9-dibromo-2,10-dinitro-6,7-dihydro-5*H*-diimidazo [1,2-a:2′,1′-c][1,4]diazepine (**2**).

Sodium bicarbonate (0.17 g, 2.0 mmol) was added to a solution of TNBI (0.314 g, 1.0 mmol) in DMF (5 mL). The 1,3-dibromopropane (0.20 g, 1.0 mmol) was added to the mixture and then heated to 90 °C with continuous stirring for 12 h. After cooling to room temperature, the reaction mixture was poured into ice water (100 mL), and the precipitate was collected and washed with deionized water. The product was obtained by recrystallization with acetonitrile and water (0.34 g, 81.1%) ^1^H NMR (500 MHz, DMSO): δ = 4.41–4.35 (m, 4H), 2.57 (p, *J* = 5.9 Hz, 2H). ^13^C NMR (126 MHz, DMSO): 144.4, 136.0, 109.7, 48.0, 25.1. IR (KBr pellet): 2988.22, 2900.6, 1525.58, 1479.74, 1444.66, 1410.04, 1359.64, 1320.36, 1288.61, 1268.01, 1214.78, 1049.07, 1017.57, 967.07, 911.58, 875.77, 834.12, 759.06, 677.31, 638.9, 589.24, 514.46, 434.95, 418.5, 403.73 cm^−1^. Elemental analysis for C_9_H_6_Br_2_N_6_O_4_ (419.88). Calculated value (%): C 25.62, H 1.43, N 19.92. Measured value (%): C 25.60, H 1.40, N 19.94.

## 4. Conclusions

In conclusion, high-density nitroimidazole-containing fused ring compound **1** and **2** were synthesized in this paper. These two compounds possess favorable thermal stability (T_d_ > 290 °C) and low mechanical sensitivity (IS > 40 J, FS > 360 N). At the same time, their dense fused ring results are a significant feature. In particular, structural compound **2** forms an organic growth twinning. The internal structure of the twinning was explored in combination with X-ray crystal diffraction. The connection between physicochemical properties and molecular structure is explored in this paper using Hirshfeld surfaces, a 2D fingerprint plot, and electrostatic potential analysis. The unique structure of the dense compound **1** and **2** possesses high stability and low mechanical sensitivity, making them special in the evolution of energetic materials.

## Figures and Tables

**Table 1:** Crystal data and structure refinement for compound **2**.

Compound	2
Temperature (K)	296
Crystal system	Monoclinic
Space group	P2_1_/c
Unit cell dimensions a orientation	14.4361 (11)
b orientation	12.6417 (9)
c orientation	13.9228 (10)
α orientation	90
β orientation	95.278 (2)
γ orientation	90
Volume	2530.1 (3)
Z	8
Density	2.216
Max. transmission	0.7456
Min. transmission	0.4363
Goodness-of-fit on F^2^	1.033
Final R indices (I > 2sigma (I))	R_1_ = 0.0321
	wR_2_ = 0.0762
R indices (all data)	R_1_ = 0.0517
	wR_2_ = 0.0827
CCDC Number	2295688

**Table 2:** Properties of compound **1** and **2**.

Compound	T_d_^1^ (°C)	IS ^2^ (J)	FS ^3^ (N)	Ρ ^4^ (g/cm^3^)
1	292.4	>40	>360	2.49
2	311.3	>40	>360	2.35
TNT ^5^	290	30	353	1.65

^1^ Temperature of decomposition (onset). ^2^ Impact sensitivity. ^3^ Friction sensitivity. ^4^ Density measured by a gas pycnometer at 25 °C. ^5^ Refs. [24,25].

## Data Availability

Data are contained within the article and supplementary materials. CCDC 2295688 contains the supplementary crystallographic data for this paper. These data can be obtained free of charge at www.ccdc.cam.ac.uk/data_request/cif, or by emailing data_request@ccdc.cam.ac.uk, or by contacting The Cambridge Crystallographic Data Centre, 12 Union Road, Cambridge CB2 1EZ, UK; Fax: +44-1223-336-033.

## Supplementary Materials

The following supporting information can be downloaded at: https://www.mdpi.com/article/10.3390/molecules29020353/s1, Experimental specifications, Detailed analysis of the crystal structure (Tables S1–S4), Methods of calculation (Scheme S1), ^1^H and ^13^C NMR spectra of compound **1** and **2** (Figures S1–S4), IR spectra of compound **1** and **2** (Figures S5 and S6). Refs. [29,30,31,32,33,34] are cited in the Supplementary Material.
