# Supplementary material for: High-Density Energetic Materials with Low Mechanical Sensitivity and Twinning Derived from Nitroimidazole Fused Ring
Source: Molecules. 2024 Jan 10;29(2):353. doi: 10.3390/molecules29020353 (PMC10819058; doi:10.3390/molecules29020353)
Supplement: Supplementary file 1 [file molecules-29-00353-s001.zip › molecules-2789248-supplementary.pdf]

molecules

Supporting Information

High-density Energetic Materials with Low Mechanical Sensitivity and Twinning Derived from Nitroimidazole Fused Ring

Yaxin Liu, Meifang Lv, Guofeng Zhang, Zhen Dong and Zhiwen Ye *

1 Experimental specifications
2 Detailed analysis of the crystal structure
3 Methods of calculation
4 ^{1}H and ^{13}C NMR spectra of 1 and 2
5 IR spectra of 1 and 2
6 References

Experimental specifications

All solvents and base chemical materials above AR purity were purchased in the regular way without further purification. The ^{1}H and ^{13}C NMR spectra were recorded on a Bruker 500 MHz Digital NMR Spectrometer operating at 500 MHz and 126 MHz separately. The chemical shifts were referenced to TMS as the zero point, and NMR spectra were plotted with deuterated DMSO as the locked field solvent. The decomposition temperature was acquired on a differential scanning calorimeter at a heating rate of 5 K/ min in a closed standard vessel of Al_2O_3 with a nitrogen flow rate of 50 mL/min. The infrared spectra of the products were documented on an Thermo Nicolet iS10 spectrometer by using KBr plates. The friction sensitivity (FS) and impact sensitivity (IS) of the compounds were measured by the BAM friction sensitivity meter and the BAM drop hammer impact sensitivity meter according to the relevant standards of the Federal Bureau of Materials Testing.

Detailed analysis of the crystal structure

A measurable transparent needle crystal (2) with dimensions of 0.220mm×0.020 mm × 0.020 mm was assessed by X-ray diffraction. Single-crystal X-ray diffraction analysis of 2 was carried out on a Bruker D8 QUEST diffractometer with microfocus Ius 2.0 Mo-Ka radiation (λ=0.71073 Å) at 296(2) K. Data reduction and absorption corrections were performed with the SAINT and SADABS software packages, [1] respectively. The structures were solved by direct methods and refined by the full matrix least-squares based on F2 using SHELXL-2018 programme package [2]. The non-hydrogen atoms were refined anisotropically. All hydrogen atoms were placed at the calculated positions and refined as riding on the parent atoms. Tables S1–S4 provide detailed information on crystallography.

Table S1. Bond lengths [Å] for 2.

No.	Atom 1	Atom 2	Distance
1	Br1	C6	1.855(3)
2	Br2	C1	1.841(3)
3	Br3	C15	1.854(3)
4	Br4	C11	1.850(3)
5	C1	C2	1.369(4)
6	C1	N1	1.373(4)
7	C2	N2	1.357(4)
8	C2	N3	1.427(4)

9	C3	N2	1.315(4)
10	C3	N1	1.375(4)
11	C3	C4	1.461(4)
12	C4	N5	1.313(4)
13	C4	N4	1.382(4)
14	C5	N5	1.361(4)
15	C5	C6	1.365(4)
16	C5	N6	1.447(4)
17	C6	N4	1.374(3)
18	C7	N4	1.472(4)
19	C7	C8	1.494(4)
20	C7	H7A	0.9700
21	C7	H7B	0.9700
22	C8	C9	1.502(5)
23	C8	H8A	0.9700
24	C8	H8B	0.9700
25	C9	N1	1.469(4)
26	C9	H9A	0.9700
27	C9	H9B	0.9700
28	C10	N8	1.352(4)
29	C10	C11	1.360(4)
30	C10	N9	1.435(4)
31	C11	N7	1.370(4)
32	C12	N8	1.315(4)
33	C12	N7	1.378(4)
34	C12	C13	1.462(4)
35	C13	N11	1.321(4)
36	C13	N10	1.370(4)
37	C14	N11	1.363(4)
38	C14	C15	1.369(4)
39	C14	N12	1.434(4)
40	C15	N10	1.367(4)
41	C16	C17B	1.392(10)
42	C16	C17A	1.430(6)
43	C16	N10	1.471(4)
44	C16	H16A	0.9700
45	C16	H16B	0.9700
46	C16	H16C	0.9700
47	C16	H16D	0.9700
48	C17A	C18	1.471(6)
49	C17A	H17A	0.9700
50	C17A	H17B	0.9700
51	C17B	C18	1.481(10)
52	C17B	H17C	0.9700
53	C17B	H17D	0.9700
54	C18	N7	1.476(4)
55	C18	H18A	0.9700
56	C18	H18B	0.9700
57	C18	H18C	0.9700

58	C18	H18D	0.9700
59	N3	O1	1.211(4)
60	N3	O2	1.216(4)
61	N6	O3	1.198(3)
62	N6	O4	1.219(3)
63	N9	O5	1.219(3)
64	N9	O6	1.219(4)
65	N12	O7B	1.21(2)
66	N12	O8A	1.22(3)
67	N12	O7A	1.24(2)
68	N12	O8B	1.25(3)

Table S2. Bond angles [°] for 2.

No.	Atom 1	Atom 2	Atom 3	Angle
1	C2	C1	N1	104.3(2)
2	C2	C1	Br2	132.5(2)
3	N1	C1	Br2	123.2(2)
4	N2	C2	C1	112.9(3)
5	N2	C2	N3	120.4(3)
6	C1	C2	N3	126.7(3)
7	N2	C3	N1	112.6(3)
8	N2	C3	C4	121.3(3)
9	N1	C3	C4	126.1(3)
10	N5	C4	N4	112.4(2)
11	N5	C4	C3	120.2(3)
12	N4	C4	C3	127.4(3)
13	N5	C5	C6	112.5(3)
14	N5	C5	N6	120.1(3)
15	C6	C5	N6	127.4(3)
16	C5	C6	N4	105.0(2)
17	C5	C6	Br1	133.9(2)
18	N4	C6	Br1	121.1(2)
19	N4	C7	C8	115.9(3)
20	N4	C7	H7A	108.300
21	C8	C7	H7A	108.300
22	N4	C7	H7B	108.300
23	C8	C7	H7B	108.300
24	H7A	C7	H7B	107.400
25	C7	C8	C9	114.6(3)
26	C7	C8	H8A	108.600
27	C9	C8	H8A	108.600
28	C7	C8	H8B	108.600
29	C9	C8	H8B	108.600
30	H8A	C8	H8B	107.600
31	N1	C9	C8	111.8(3)
32	N1	C9	H9A	109.300
33	C8	C9	H9A	109.300
34	N1	C9	H9B	109.300
35	C8	C9	H9B	109.300

36	H9A	C9	H9B	107.900
37	N8	C10	C11	112.3(3)
38	N8	C10	N9	120.8(3)
39	C11	C10	N9	126.9(3)
40	C10	C11	N7	105.1(2)
41	C10	C11	Br4	132.2(2)
42	N7	C11	Br4	122.7(2)
43	N8	C12	N7	111.8(3)
44	N8	C12	C13	120.6(3)
45	N7	C12	C13	127.6(3)
46	N11	C13	N10	112.2(2)
47	N11	C13	C12	119.7(3)
48	N10	C13	C12	128.0(3)
49	N11	C14	C15	112.4(3)
50	N11	C14	N12	120.2(3)
51	C15	C14	N12	127.4(3)
52	N10	C15	C14	104.6(2)
53	N10	C15	Br3	122.6(2)
54	C14	C15	Br3	132.8(2)
55	C17B	C16	N10	120.1(5)
56	C17A	C16	N10	115.3(3)
57	C17A	C16	H16A	108.500
58	N10	C16	H16A	108.500
59	C17A	C16	H16B	108.500
60	N10	C16	H16B	108.500
61	H16A	C16	H16B	107.500
62	C17B	C16	H16C	107.300
63	N10	C16	H16C	107.300
64	C17B	C16	H16D	107.300
65	N10	C16	H16D	107.300
66	H16C	C16	H16D	106.900
67	C16	C17A	C18	120.9(5)
68	C16	C17A	H17A	107.100
69	C18	C17A	H17A	107.100
70	C16	C17A	H17B	107.100
71	C18	C17A	H17B	107.100
72	H17A	C17A	H17B	106.800
73	C16	C17B	C18	122.9(8)
74	C16	C17B	H17C	106.600
75	C18	C17B	H17C	106.600
76	C16	C17B	H17D	106.600
77	C18	C17B	H17D	106.600
78	H17C	C17B	H17D	106.600
79	C17A	C18	N7	115.8(3)
80	N7	C18	C17B	115.4(4)
81	C17A	C18	H18A	108.300
82	N7	C18	H18A	108.300
83	C17A	C18	H18B	108.300
84	N7	C18	H18B	108.300

85	H18A	C18	H18B	107.400
86	N7	C18	H18C	108.400
87	C17B	C18	H18C	108.400
88	N7	C18	H18D	108.400
89	C17B	C18	H18D	108.400
90	H18C	C18	H18D	107.500
91	C1	N1	C3	106.5(2)
92	C1	N1	C9	125.6(3)
93	C3	N1	C9	127.9(2)
94	C3	N2	C2	103.7(2)
95	O1	N3	O2	123.4(3)
96	O1	N3	C2	117.5(3)
97	O2	N3	C2	119.1(3)
98	C6	N4	C4	106.0(2)
99	C6	N4	C7	121.6(2)
100	C4	N4	C7	132.4(2)
101	C4	N5	C5	104.1(2)
102	O3	N6	O4	124.2(3)
103	O3	N6	C5	118.9(3)
104	O4	N6	C5	117.0(3)
105	C11	N7	C12	106.3(2)
106	C11	N7	C18	123.3(2)
107	C12	N7	C18	130.5(3)
108	C12	N8	C10	104.5(2)
109	O5	N9	O6	123.9(3)
110	O5	N9	C10	117.6(3)
111	O6	N9	C10	118.4(2)
112	C15	N10	C13	107.0(2)
113	C15	N10	C16	123.7(3)
114	C13	N10	C16	129.4(3)
115	C13	N11	C14	103.8(2)
116	O8A	N12	O7A	121.9(18)
117	O7B	N12	O8B	128.(2)
118	O7B	N12	C14	116.1(14)
119	O8A	N12	C14	118.4(15)
120	O7A	N12	C14	119.5(11)
121	O8B	N12	C14	115.8(16)

Table S3. Torsion angles [°] for 2.

No.	Atom 1	Atom 2	Atom 3	Atom 4	Torsion angle
1	N1	C1	C2	N2	-0.2(3)
2	Br2	C1	C2	N2	179.5(2)
3	N1	C1	C2	N3	-179.2(3)
4	Br2	C1	C2	N3	0.4(5)
5	N2	C3	C4	N5	-13.0(4)
6	N1	C3	C4	N5	165.6(3)
7	N2	C3	C4	N4	166.5(3)

8	N1	C3	C4	N4	-14.9(5)
9	N5	C5	C6	N4	-0.3(4)
10	N6	C5	C6	N4	178.2(3)
11	N5	C5	C6	Br1	-179.9(2)
12	N6	C5	C6	Br1	-1.4(5)
13	N4	C7	C8	C9	61.6(4)
14	C7	C8	C9	N1	-82.7(4)
15	N8	C10	C11	N7	0.1(3)
16	N9	C10	C11	N7	179.1(3)
17	N8	C10	C11	Br4	178.2(2)
18	N9	C10	C11	Br4	-2.8(5)
19	N8	C12	C13	N11	2.3(4)
20	N7	C12	C13	N11	-179.7(3)
21	N8	C12	C13	N10	-173.6(3)
22	N7	C12	C13	N10	4.4(5)
23	N11	C14	C15	N10	-0.3(4)
24	N12	C14	C15	N10	179.9(3)
25	N11	C14	C15	Br3	-179.6(2)
26	N12	C14	C15	Br3	0.6(5)
27	N10	C16	C17A	C18	71.2(6)
28	N10	C16	C17B	C18	-60.3(11)
29	C16	C17A	C18	N7	-65.0(6)
30	C16	C17B	C18	N7	63.7(10)
31	C2	C1	N1	C3	0.1(3)
32	Br2	C1	N1	C3	-179.7(2)
33	C2	C1	N1	C9	179.9(3)
34	Br2	C1	N1	C9	0.2(4)
35	N2	C3	N1	C1	0.1(3)
36	C4	C3	N1	C1	-178.6(3)
37	N2	C3	N1	C9	-179.8(3)
38	C4	C3	N1	C9	1.5(5)
39	C8	C9	N1	C1	-133.2(3)
40	C8	C9	N1	C3	46.6(4)
41	N1	C3	N2	C2	-0.2(3)
42	C4	C3	N2	C2	178.6(3)
43	C1	C2	N2	C3	0.2(3)
44	N3	C2	N2	C3	179.3(3)
45	N2	C2	N3	O1	173.3(3)
46	C1	C2	N3	O1	-7.7(5)
47	N2	C2	N3	O2	-6.9(5)
48	C1	C2	N3	O2	172.1(3)
49	C5	C6	N4	C4	1.1(3)
50	Br1	C6	N4	C4	-179.3(2)
51	C5	C6	N4	C7	179.4(3)
52	Br1	C6	N4	C7	-1.0(4)
53	N5	C4	N4	C6	-1.6(3)
54	C3	C4	N4	C6	178.8(3)
55	N5	C4	N4	C7	-179.6(3)
56	C3	C4	N4	C7	0.8(5)

57	C8	C7	N4	C6	166.8(3)
58	C8	C7	N4	C4	-15.4(5)
59	N4	C4	N5	C5	1.4(3)
60	C3	C4	N5	C5	-179.0(3)
61	C6	C5	N5	C4	-0.7(4)
62	N6	C5	N5	C4	-179.3(3)
63	N5	C5	N6	O3	8.0(5)
64	C6	C5	N6	O3	-170.4(4)
65	N5	C5	N6	O4	-171.0(3)
66	C6	C5	N6	O4	10.6(5)
67	C10	C11	N7	C12	-1.0(3)
68	Br4	C11	N7	C12	-179.3(2)
69	C10	C11	N7	C18	-179.6(3)
70	Br4	C11	N7	C18	2.1(4)
71	N8	C12	N7	C11	1.6(3)
72	C13	C12	N7	C11	-176.5(3)
73	N8	C12	N7	C18	-179.9(3)
74	C13	C12	N7	C18	1.9(5)
75	C17A	C18	N7	C11	-160.6(4)
76	C17B	C18	N7	C11	147.7(6)
77	C17A	C18	N7	C12	21.1(6)
78	C17B	C18	N7	C12	-30.5(7)
79	N7	C12	N8	C10	-1.6(3)
80	C13	C12	N8	C10	176.7(3)
81	C11	C10	N8	C12	0.9(3)
82	N9	C10	N8	C12	-178.2(3)
83	N8	C10	N9	O5	173.4(3)
84	C11	C10	N9	O5	-5.5(5)
85	N8	C10	N9	O6	-4.6(4)
86	C11	C10	N9	O6	176.4(3)
87	C14	C15	N10	C13	0.0(3)
88	Br3	C15	N10	C13	179.4(2)
89	C14	C15	N10	C16	179.5(3)
90	Br3	C15	N10	C16	-1.1(4)
91	N11	C13	N10	C15	0.3(3)
92	C12	C13	N10	C15	176.4(3)
93	N11	C13	N10	C16	-179.2(3)
94	C12	C13	N10	C16	-3.1(5)
95	C17B	C16	N10	C15	-155.0(6)
96	C17A	C16	N10	C15	150.0(4)
97	C17B	C16	N10	C13	24.5(8)
98	C17A	C16	N10	C13	-30.6(6)
99	N10	C13	N11	C14	-0.5(3)
100	C12	C13	N11	C14	-176.9(3)
101	C15	C14	N11	C13	0.5(4)
102	N12	C14	N11	C13	-179.7(3)
103	N11	C14	N12	O7B	13.(2)
104	C15	C14	N12	O7B	-167.(2)
105	N11	C14	N12	O8A	168.(2)

106	C15	C14	N12	O8A	-12.(2)
107	N11	C14	N12	O7A	-17.(4)
108	C15	C14	N12	O7A	163.(4)
109	N11	C14	N12	O8B	-170.(3)
110	C15	C14	N12	O8B	10.(3)

Table S4. Hydrogen bonds for 2 [Å and °].

No.	Donor	H atom	Acceptor	Dist. D-H	Dist. H-A	Dist. D-A	Angle D-H-A
1	C7	H7A	Br1	0.97	2.49	2.97(3)	110.3
2	C8	H8A	Br1	0.97	3.00	3.824(3)	144.0
3	C8	H8A	O6	0.97	2.64	3.470(5)	143.4
4	C8	H8B	O5	0.97	2.57	3.250(4)	127.0
5	C9	H9B	Br2	0.97	2.73	3.254(3)	114.1
6	C9	H9B	O5	0.97	2.42	3.166(4)	133.0
7	C16	Br1	Br3	0.97	2.73	3.200(4)	110.2
8	C16	Br1	O3	0.97	2.39	3.078(4)	127.3
9	C16	Br1	O2	0.97	2.55	3.34(4)	139.1
10	C18	Br1	Br3	0.97	3.06	3.823(4)	136.2
11	C18	Br1	Br4	0.97	2.72	3.196(3)	110.7
12	C18	Br1	O1	0.97	2.39	3.013(4)	121.3

1: -x+1,y-1/2,-z+1/2; 2: x,y-1,z; 3: x-1,y,z; 4: -x,-y+1,-z+1;.

5: -x+1,-y+1,-z+1; 6: -x,y+1/2,-z+1/2; 7: x-1,y+1,z.

Methods of calculation

The calculations were carried out by using the *Gaussian 09* suite of programs.[3]The fundamental geometrical optimisation and frequency analysis were performed at the level of the Becke three parameter, Lee-Yan-Parr (B3LYP)[4,5] functional with the 6- 311++G** basis set.[6] All optimised structures were characterised as the local energy minima of the potential surface without any imaginary frequencies. Atomisation energies were calculated by the CBS-4M.[7] All optimised structures were described as true local energy minima on the potential energy surface with no imaginary frequencies.

The heats of generation (HOF) of compounds were predicted by using the hybrid DFTB3LYP method with the 6-311++G** basis set through designed isodesmic reactions. In the case of the application of the Bond Separation Reaction (BSR) rules, the isostructural reaction processes was used, meaning that the number of each form of bond is conserved. The molecule was broken down into a set of two heavy-atom molecules containing the same component bonds. The isodesmic reactions used to derive the HOF of compound 1 and 2 are shown in Scheme S1.

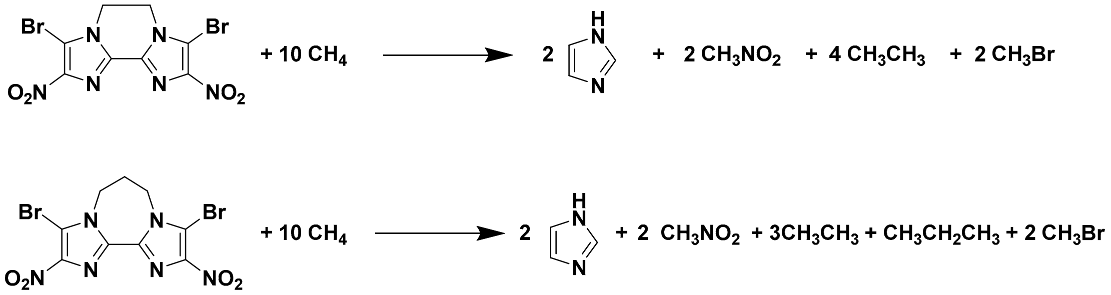

Scheme S1. Isodesmic reaction for calculating heat of formation for 1 and 2.

^{1}H and ^{13}C NMR spectra of 1 and 2

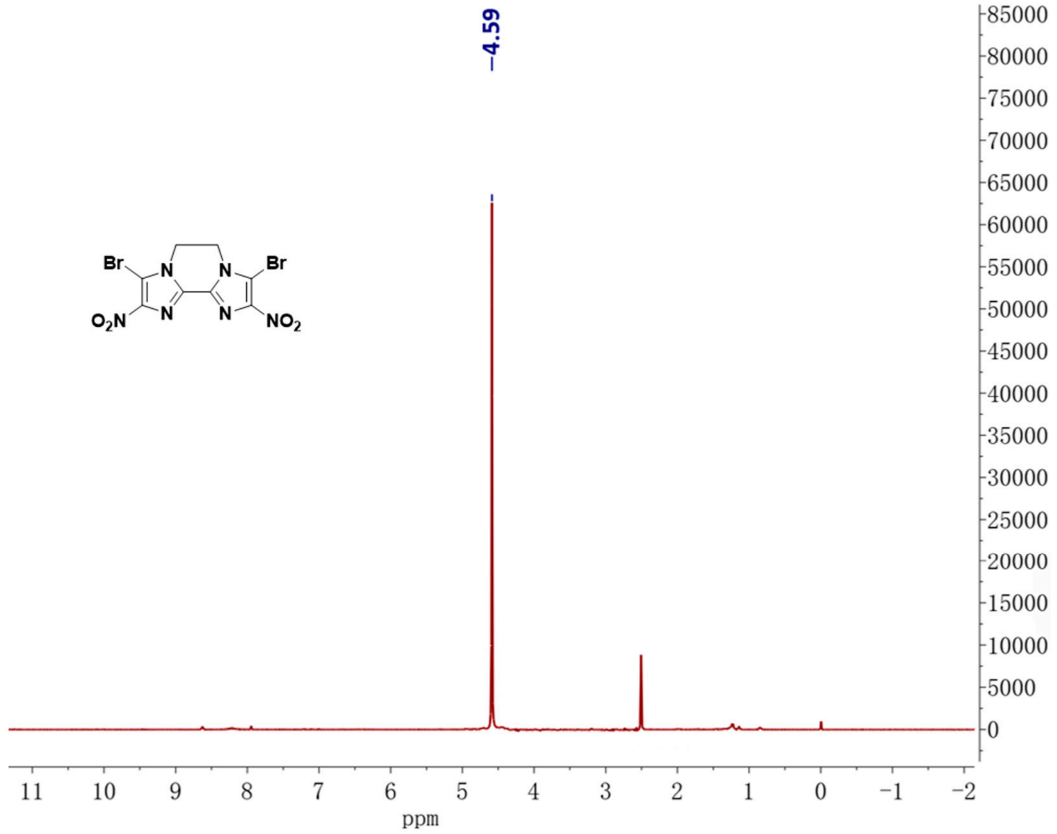

Figure S1. ^{1}H NMR spectrum of 1.

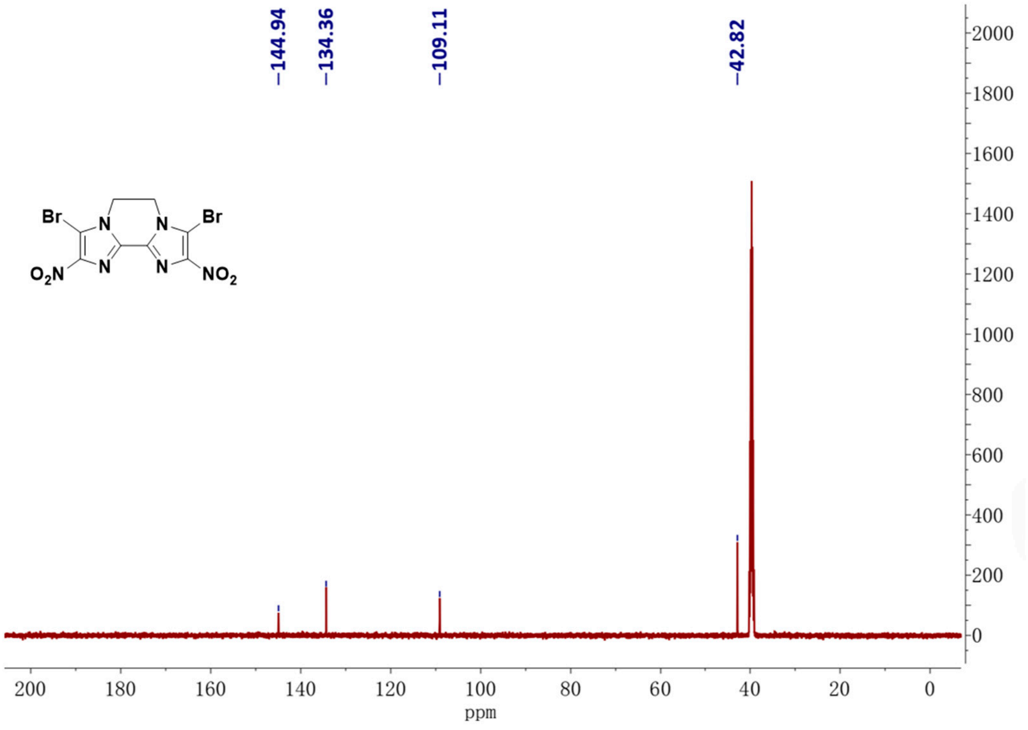

Figure S2. ^{13}C NMR spectrum of 1.

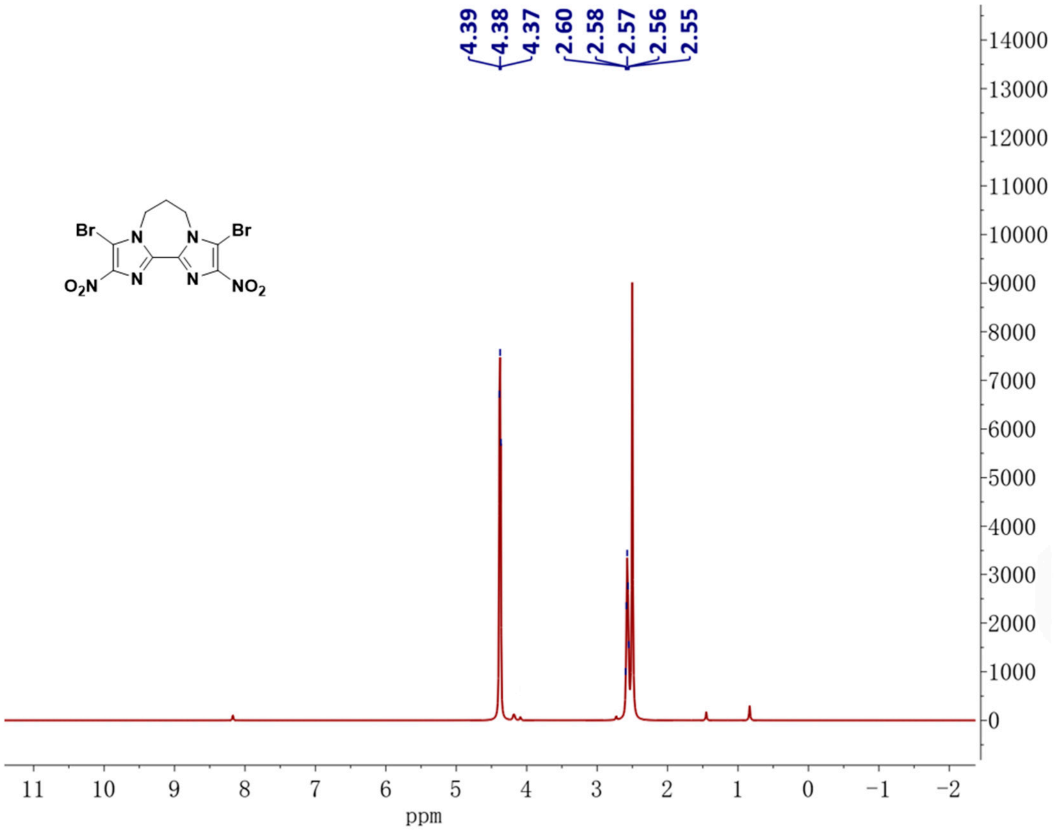

Figure S3. ^{1}H NMR spectrum of 2.

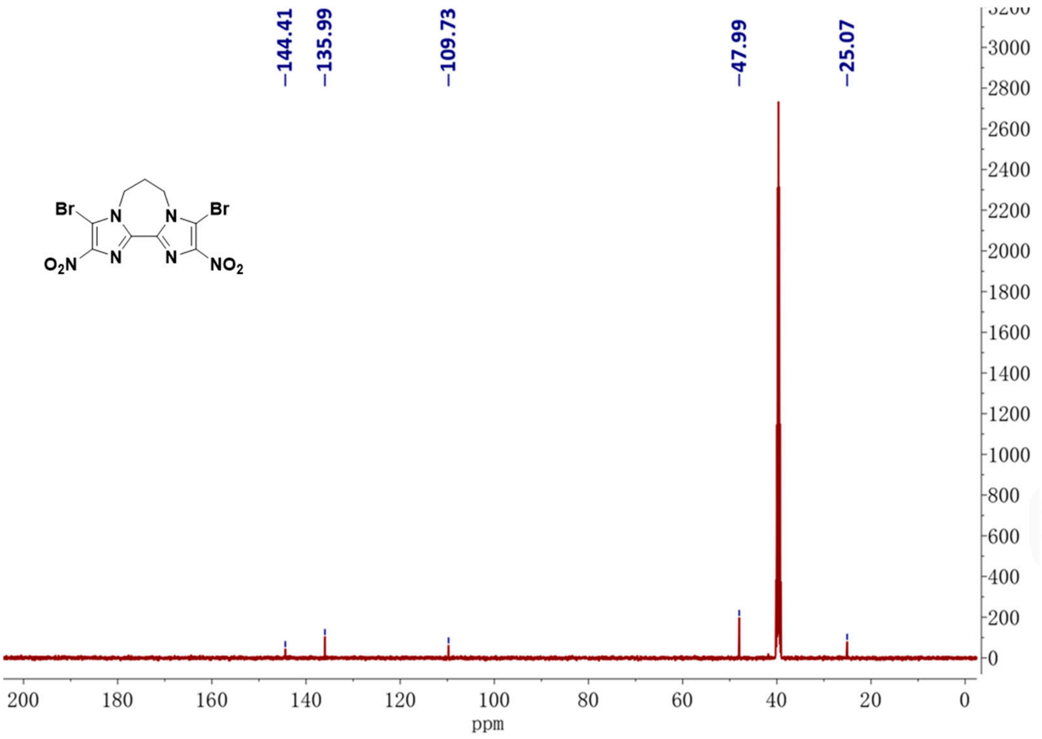

Figure S4. ^{13}C NMR spectrum of 2.

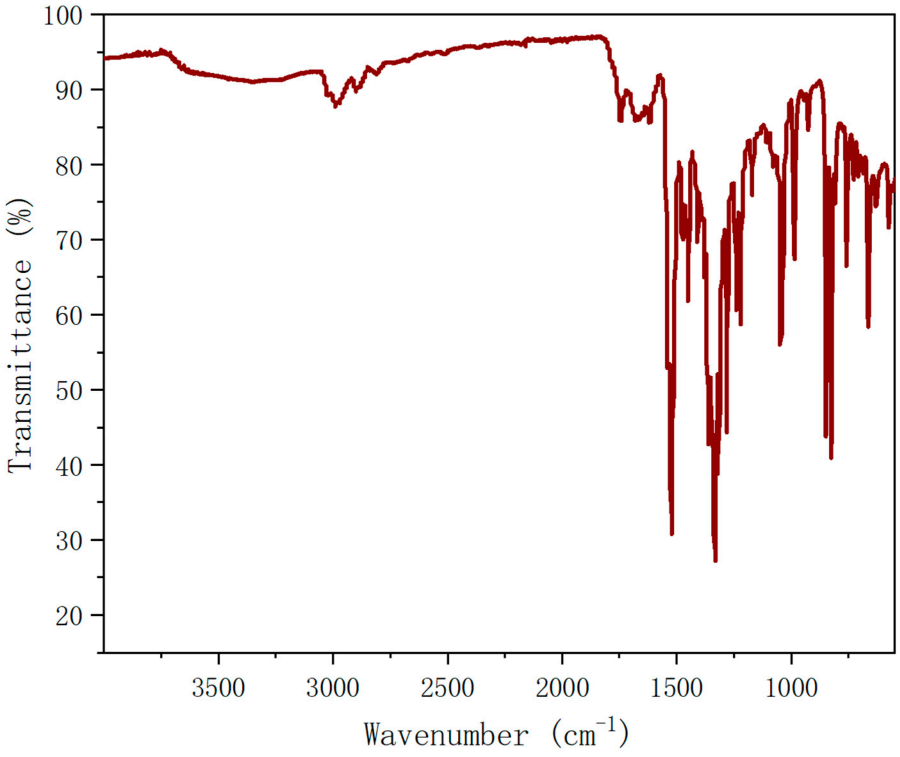

Figure S5. IR spectrum of 1.

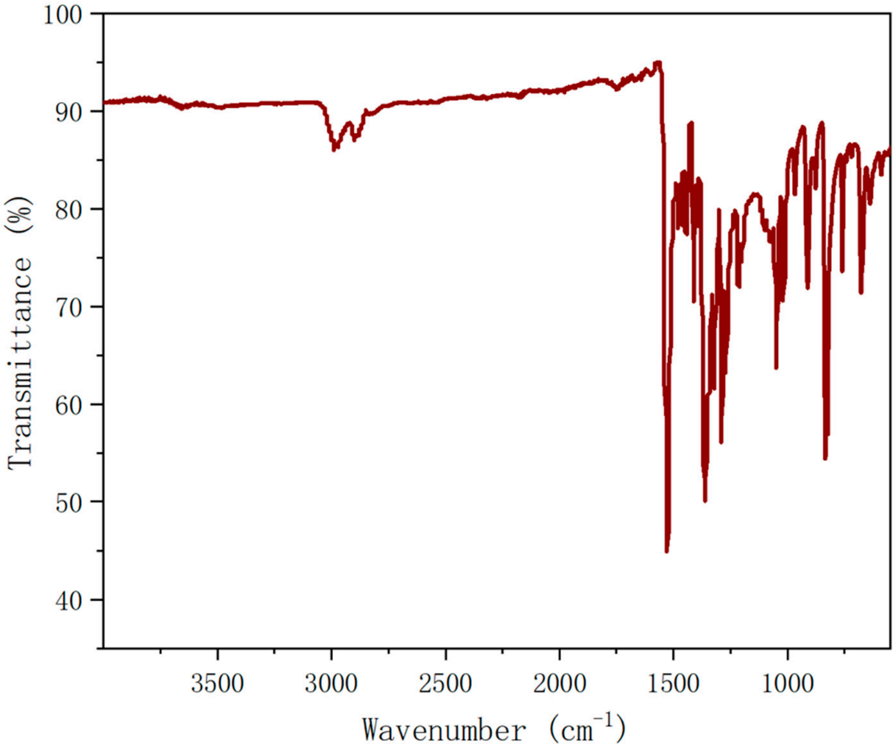

Figure S6. IR spectrum of 2.

References

1. Bruker, APEX3 *v2019.1*, Bruker Nano, Inc., Madison, Wisconsin, WI, USA, 20019.
2. Sheldrick, G. M. SHELXT - Integrated Space-Group and Crystal-Structure Determination. *Acta Crystallogr. A Found. Adv.* **2015**, *71*, 3–8.
3. Frisch, M. J.; Trucks, G. W.; Schlegel, H. B.; Scuseria, G. E.; Robb, M. A.; Cheeseman, J. R.; Scalmani, G.; Barone, V.; Mennucci, B.; Petersson, G. A.; Nakatsuji, H.; Caricato, M.; Li, X.; Hratchian, H. P.; Izmaylov, A. F.; Bloino, J.; Zheng, G.; Sonnenberg, J. L.; Hada, M.; Ehara, M.; Toyota, K.; Fukuda, R.; Hasegawa, J.; Ishida, M.; Nakajima, T.; Honda, Y.; Kitao, O.; Nakai, H.; Vreven, T.; Montgomery, J. A.; Peralta, J. E.; Ogliaro, F.; Bearpark, M.; Heyd, J. J.; Brothers, E.; Kudin, K. N.; Staroverov, V. N.; Kobayashi, R.; Normand, J.; Raghavachari, K.; Rendell, A.; Burant, J. C.; Iyengar, S. S.; Tomasi, J.; Cossi, M.; Rega, N.; Millam, J. M.; Klene, M.; Knox, J. E.; Cross, J. B.; Bakken, V.; Adamo, C.; Jaramillo, J.; Gomperts, R.; Stratmann, R. E.; Yazyev, O.; Austin, A. J.; Cammi, R.; Pomelli, C.; Ochterski, J. W.; Martin, R. L.; Morokuma, K.; Zakrzewski, V. G.; Voth, G. A.; Salvador, P.; Dannenberg, J. J.; Dapprich, S.; Daniels, A. D.; Farkas, O.; Foresman, J. B.; Ortiz, J. V.; Cioslowski, J.; Fox, D. J.; Gaussian 09, *Revision A.1*, Gaussian, Inc., Wallingford CT, 2009.
4. Becke, A. D. Density-Functional Thermochemistry. III. The Role of Exact Exchange. *J. Chem. Phys.* **1993**, *98*, 5648–5652.
5. Stephens, P. J.; Devlin, F. J.; Chabalowski, C. F.; Frisch, M. J.Ab Initio Calculation of Vibrational Absorption and Circular Dichroism Spectra Using Density Functional Force Fields *J. Phys. Chem.* **1994**, *98*, 11623–11627.
6. Hariharan, P. C.; Pople, J. A. The Influence of Polarization Functions on Molecular Orbital Hydrogenation Energies. *Theor. Chim. Acta.* **1973**, *28*, 213–222.
7. Ochterski, J. W.; Petersson, G. A.; Montgomery, J. A. A Complete Basis Set Model Chemistry. V. Extensions to Six or More Heavy Atoms. *J. Chem. Phys.* **1996**, *104*, 2598–2619.
